# Dynamics of viral self-assembly, viral genome packaging, and virus-cell interactions studied by optical tweezers

**DOI:** 10.1007/s00249-026-01843-6

**Published:** 2026-04-30

**Authors:** Yanping Gong, Dorus T. Harmsen, Wouter H. Roos

**Affiliations:** Moleculaire Biofysica, Zernike Instituut, Rijksuniversiteit Groningen, Groningen, The Netherlands

**Keywords:** Viruses, Optical tweezers, Single-molecule biophysics, Supramolecular self-assembly, Nucleic acid packaging, Viral infection, DNA–protein interactions, Molecular motors, Physical virology

## Abstract

Viruses are complex supramolecular assemblies that propagate their genetic material from cell to cell, thereby relying on host cell mechanisms. Employing a combination of passive and active strategies, they efficiently package, transport and release nucleic acids. While structural and biochemical techniques offer insights into certain, static aspects of the viral life cycle, recent advancements in biophysical approaches now allow for direct measurement of their inherent dynamic activities in the research field commonly referred to as physical virology. One of these methods is optical tweezers, enabling the precise measurement of force and position at the single-molecule level over time. Over the past decades, the ability to optically trap beads and to manipulate biomolecules has revolutionised medical and biophysical research. In this paper, we provide a comprehensive analysis of optical tweezers, exploring its integration with imaging modalities and review its diverse applications in the study of viruses and viral components. In particular we focus on studies that use optical tweezers to study virus-cell interactions, genome packaging using molecular motors and co-assembly of viral assembly proteins with their nucleic acid.

## Introduction

Unlike bulk methods that are used to study viruses, research on viruses using optical tweezers is still in its infancy. This is due to the relatively recent development of single-molecule approaches for the study of biological specimens. Currently, there is significant interest in not only real-time characterisation of viral mechanochemical and biophysical functions, but also for leveraging this knowledge for nanotechnological applications. In this review we will first briefly introduce viruses and optical tweezers and then discuss applications of this technique to study viruses at the single particle level.

## Background

### Studying viruses

The virus life cycle is a cyclic process where an infectious viral particle (virion) introduces its genome into a host cell, leading to the formation and release of new virions, which then infect other cells. Virions formed within a host cell are generally dynamic and metastable; they are robust enough to protect the viral genome outside the cell while also being able to undergo structural changes and perform mechanochemical actions necessary for infecting other cells. The complexity of viral structures necessitates the use of various techniques to study both their static and dynamic aspects. Next to bulk biochemical approaches, common static methods include electron microscopy (EM), X-ray crystallography, nuclear magnetic resonance spectroscopy (NMR), mass spectrometry (MS), and other spectroscopic techniques (Mateu 2013, Wörner et al. 2021, Lecoq et al. 2020). On the other hand, dynamic viral processes are typically investigated by fluorescence microscopy (Arista-Romero et al. 2021, Liu et al. 2020) or by using single-molecule/single particle methods (Bruinsma et al. 2021, Kiss et al. 2020). The latter encompass tools such as atomic force microscopy (AFM), optical tweezers, magnetic tweezers and interferometric scattering (Marchetti et al. 2016, Seifert et al. 2020, Garmann et al. 2019, Buzón et al. 2020, Arias-Gonzalez 2024). These single-molecule techniques enable the observation of viruses in action, tracking the packaging of nucleic acids into the viral capsid, their self-assembly and their mechanics, providing unique insights into the physical principles underlying viral life cycle processes.

### Optical tweezers

**Fig. 1:**
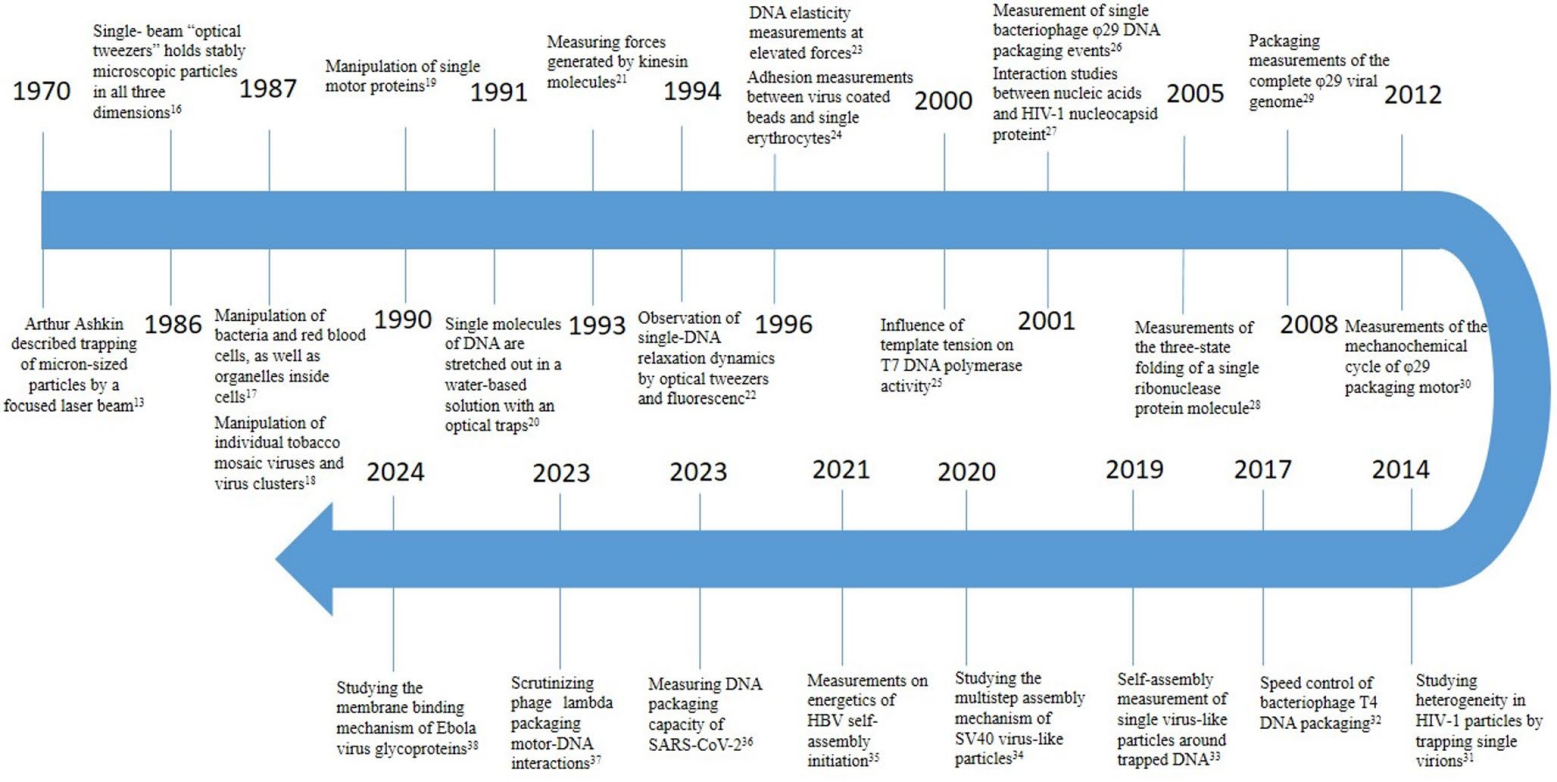
Milestones of optical tweezers in biophysics. A few general milestones are mentioned, but the majority of milestones is focused on physical virology approaches. 1970 Arthur Ashkin described trapping of micron-sized particles by a focused laser beam (Ashkin 1970). 1986 Single- beam “optical tweezers” holds stably microscopic particles in all three dimensions (Ashkin et al. 1986). 1987 Manipulation of bacteria and red blood cells, as well as organelles inside cells (Ashkin et al. 1987). 1987 Manipulation of individual tobacco mosaic viruses and virus clusters (Ashkin and Dziedzic 1987). 1990 Manipulation of single motor proteins (Block et al. 1990). 1991 Single molecules of DNA are stretched out in a water-based solution with an optical trap (Chu 1991). 1993 Measuring forces generated by kinesin molecules (Svoboda et al. 1993). 1994 Observation of single-DNA relaxation dynamics by optical tweezers and fluorescence microscopy (Perkins et al. 1994). 1996 DNA elasticity measurements at elevated forces (Smith et al. 1996). 1996 Adhesion measurements between virus coated beads and single erythrocytes (Mammen et al. 1996). 2000 Influence of template tension on T7 DNA polymerase activity (Wuite et al. 2000). 2001 Measurement of single bacteriophage $\:\phi\:$29 DNA packaging events (Smith et al. 2001). 2001 Interaction studies between nucleic acids and HIV-1 nucleocapsid protein (Williams et al. 2001). 2005 Measurements of the three-state folding of a single ribonuclease protein molecule (Cecconi et al. 2005). 2008 Packaging measurements of the complete phage $\:\phi\:$29 viral genome (Rickgauer et al. 2008). 2012 Measurements of the mechanochemical cycle of phage $\:\phi\:$29 packaging motor (Chistol et al. 2012). 2014 Studying heterogeneity in HIV-1 particles by trapping single virions (Pang et al. 2014). 2017 Speed control of bacteriophage T4 DNA packaging (Lin et al. 2017). 2019 Self-assembly measurement of single virus-like particles around trapped DNA (Marchetti et al. 2019). 2020 Studying the multistep assembly mechanism of SV40 virus-like particles (Rosmalen et al. 2020). 2021 Measurements on energetics of HBV self-assembly initiation (Buzón et al. 2021). 2023 Measuring DNA packaging capacity of SARS-CoV-2 (Morse et al. 2023). 2023 Scrutinizing phage lambda packaging motor-DNA interactions (Rawson et al. 2023). 2024 Studying the membrane binding mechanism of Ebola virus glycoproteins (Vaknin et al. 2024)

Optical tweezers is a technique that uses the optical forces generated by a change in momentum of refracted photons to achieve optical trapping of micron-sized, transparent beads. This method is now widely used in the field of biophysics (Ashkin 1970, Bustamante et al. 2021). Throughout the history of optical tweezers, from Arthur Ashkin describing in 1970 how micron-sized particles can be trapped in liquid (Ashkin 1970), through the award of the Nobel Prize in Physics to him in 2018, and continuing with ongoing research today, optical tweezers have been used to study a variety of molecular biophysical processes (Bustamante et al. 2021, Ashkin 2000). The strength of optical tweezers is its high resolution in terms of force and position as well as their versatility and dynamic range. Over the past 50 years, optical tweezers-based research has driven significant advances in the fields of biophysics and virology (Fig. 1). As a single-molecule technique, it provided and provides new insights into the viral life cycle by allowing researchers to observe and manipulate the interactions of individual molecules, thereby revealing detailed mechanisms of viral processes.

**Fig. 2:**
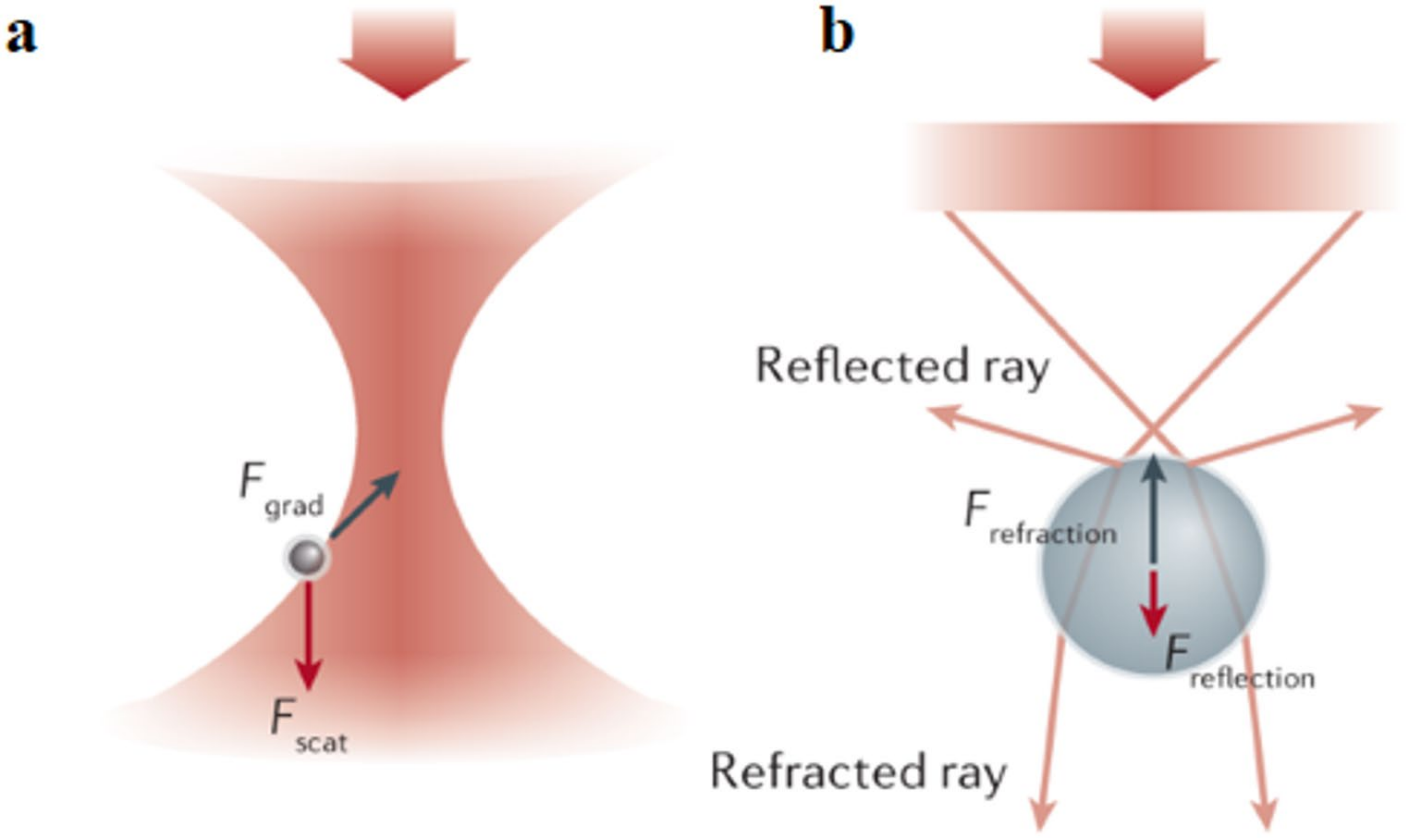
Forces acting on a dielectric sphere interacting with light, with the incident light beam focused by a high-numerical aperture lens (Bustamante et al. 2021). (**a**) a particle much smaller than the wavelength of light can be described as a Rayleigh scatterer. The optical forces that the particle experiences are here depicted as a scattering force (F_scat_, red arrow) that pushes the particle in the direction of light propagation, and a gradient force (F_grad_, black arrow) that pulls it toward the focal point of the beam. (**b**) the same forces act on a particle larger than the wavelength of light, but here these forces can be described using ray optics. The particle acts as a refractive object that refracts incoming rays (pink arrows) focused by a high-NA lens. These deflections change the light’s momentum, producing equal and opposite forces on the bead. Reflected rays push the bead forward (F_reflection_, red arrow), while refracted rays pull it toward the focus (F_refraction_, black arrow). Reproduced from ref (Bustamante et al. 2021) with permission

When a transparent bead interacts with a focused beam of light, it experiences two distinct forces: the gradient force (*F*_*grad*_) and the scattering force (*F*_*scat*_) (Rohrbach and Stelzer 2001).The gradient force pulls the object towards regions of higher light intensity and is directly proportional to the gradient of light intensity. The scattering force arises from the absorption and scattering of light by the bead. This force typically pushes the particle in the direction of the incident light (Ashkin 1970, Bustamante et al. 2021). In a highly focused laser beam, the gradient force can overcome the scattering force, leading to stable trapping of the object in three dimensions (Bustamante et al. 2021, Ashkin 2000). Figure 2 shows a schematic of trapping a bead, the involved forces and how the incident light is reflected and refracted.

In typical optical tweezers experiments, the biological sample of interest - such as for instance a small virus or a DNA molecule - is too small to be trapped directly by the focused laser light. Instead, it is tethered to a micron-sized bead, typically composed of polystyrene or silica and ranging from ~ 0.2 to 5 μm in diameter (Neuman and Block 2004). Linker molecules, e.g. biotin-streptavidin or double-stranded DNA fragments known as “molecular handles” are commonly used to attach the molecule of interest to the beads (Cecconi et al. 2008, Hashemi Shabestari et al. 2017).

To properly discuss the physics of optical tweezers, a distinction is made between objects that are smaller than the wavelength of light, called Rayleigh scatterers, and objects that are larger than the wavelength of the light, which behave as refractive objects (Arias-Gonzalez 2024, Bustamante et al. 2021, Rohrbach and Stelzer 2001). The scattering force acting on a Rayleigh scatterer can be described by (Bustamante et al. 2021).

$$\:{ \overset{\lower0.5em\hbox{$\smash{\scriptscriptstyle\rightharpoonup}$}} {F} }_{scat}=\frac{{n}_{m}\sigma\:}{c}\langle\overset{\lower0.5em\hbox{$\smash{\scriptscriptstyle\rightharpoonup}$}} {S} \rangle$$

where $$\:\sigma\:$$ is the extinction cross-section, *n*_*m*_ is the refractive index, *c* is the speed of light and $$\: \overset{\lower0.5em\hbox{$\smash{\scriptscriptstyle\rightharpoonup}$}} {S}$$ is the Poynting vector, which is a vector that is proportional to the momentum of the light and points in the direction of light propagation (Bustamante et al. 2021, Rohrbach and Stelzer 2001). The gradient force acting on a Rayleigh scatterer is given by (Bustamante et al. 2021)

$$\:{ \overset{\lower0.5em\hbox{$\smash{\scriptscriptstyle\rightharpoonup}$}} {F} }_{gradient}=\:\frac{\alpha\:}{2c{n}_{m}{\epsilon}_{0}}\nabla\:I$$

where $\:\alpha\:$ is the polarizability of the particle and $\:{\epsilon}_{0}$ is the permittivity of vacuum. The gradient force is thus directly proportional to the gradient of the intensity and points towards the point of highest intensity. Most commonly used laser beams have a Gaussian intensity distribution, so that the gradient force attracts particles to the centre of the beam. By generating a tight focus, the gradient force also pulls the particle towards the focal point, trapping the particle in three directions. It should be appreciated that this is a simplified explanation, more formal descriptions are available in the literature (Bustamante et al. 2021, Rohrbach and Stelzer 2001).

For objects larger than the wavelength of the light, it is more intuitive to discuss the physics based on geometrical ray optics. Often, the beads used in optical tweezers are comparable in size or larger than the wavelength of the lasers. Objects encountering a laser beam either refract or reflect a portion of the light. Both of these are paired with a change in the direction of the light and, therefore, a change in momentum. The law of conservation of momentum then requires a counteracting force in the opposite direction (see Fig. 2). A stable optical trap is achieved once all the forces on the object counteract each other, which occurs inside the focus. Generating a tight focus with a high-NA objective lens is thus essential to obtain a stable trap (Bustamante et al. 2021).

In a real optical tweezers instrument, the laser light is focused by a microscope objective lens into the sample chamber to generate an optical trap. The transmitted light is collected by a condenser lens and directed to a position sensitive detector. Stably trapping a single molecule in a laser beam is very challenging, especially when many of the same molecules are present. Instead, most optical tweezers setups use relatively large beads to which molecules can be attached. Mechanical force can be exerted on the system by manipulating the bead through movement of the trap. Additionally, forces acting on the bead can be accurately measured since any force represents a change in momentum of the bead and, through conservation of momentum, induces a deflection in the trapping laser beam. This signal is recorded with a position-sensitive photodetector and can, through careful calibration, be detected as force or movement. Beads in an optical trap experience a spring-like force (that is, for slow deformations, the force can be reasonably described by Hooke’s law, $\:F\:=\:-kx$, where k is the spring constant and x the distance from equilibrium), with spring constants on the order of 0.1 pN/nm. Detecting and quantifying biophysical processes requires careful calibration of the instrument. The most common calibration methods include Brownian motion calibration and viscous drag calibration (Bustamante et al. 2021).

### Experimental approaches

**Fig. 3:**
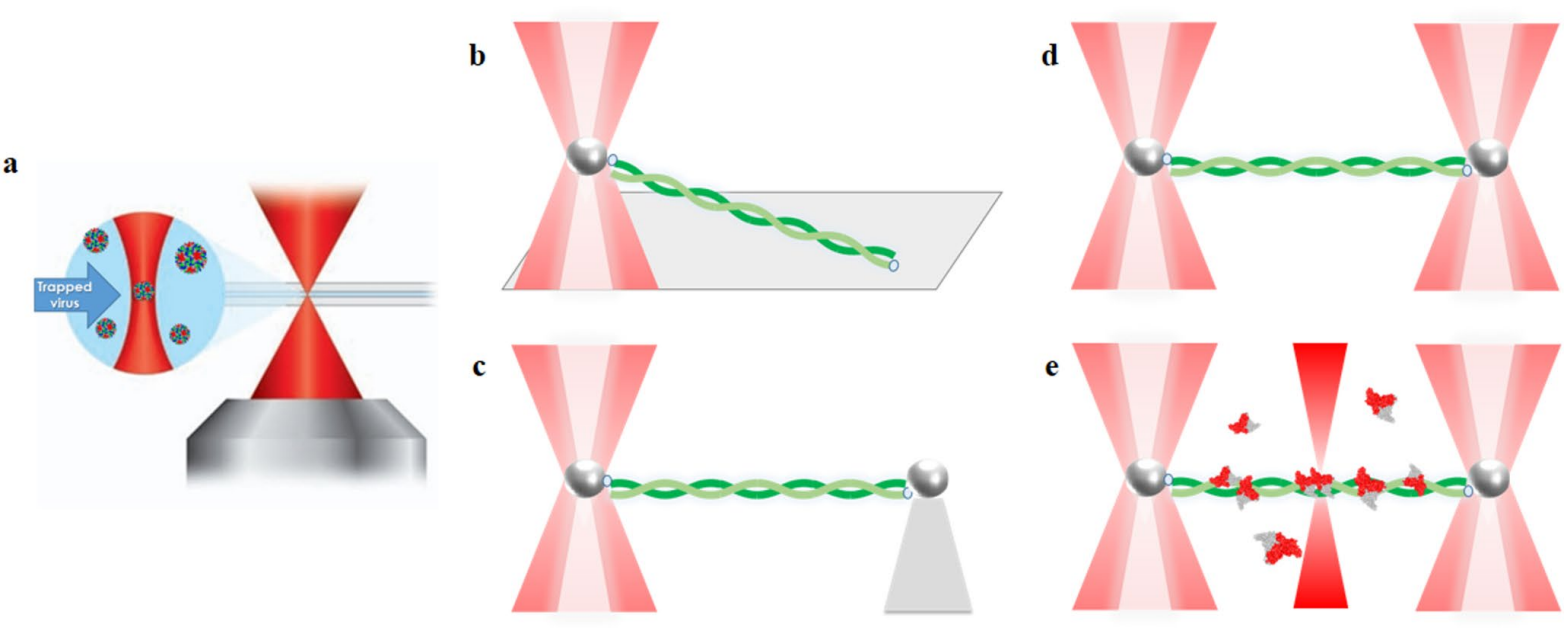
Experimental geometries of optical tweezers with virus and nucleic acid tethers. **a**) a single virus is trapped by an infrared laser focused at the centre of the sample chamber. **b**, **c**)Single-trap OT setups for nucleic acid tethers on a glass surface (**b**) or micropipette (**c**).;**d**) Dual trap optical tweezers setup for trapping nucleic acid; **e**) Dual-trap optical tweezers setup where proteins are imaged with confocal fluorescence microscopy. Inspired by ref. (Heller et al. 2014)

Most early experiments with optical tweezers were performed with a single trap. Large viruses can be trapped directly into a focused laser beam or a bead coated with viruses is trapped. Alternatively, a nucleic acid molecule is attached to a bead (Fig. 3). This can for instance be done by covalently attaching biotin molecules to the nucleic acid, which strongly binds to streptavidin molecules on a bead. The molecules are then tethered at the other end to a glass coverslip or to another bead sucked into a micropipette (Bustamante et al. 2021, Smith et al. 2001, Heller et al. 2014). Next to single optical trap set-ups, dual optical traps, where two beads are trapped in separate optical traps have become common.

Dual optical traps can for instance be made by splitting the laser beam in two parts or with an acousto-optic deflector (AOD). An AOD is a device where sound waves can be used to quickly deflect a light beam (Bustamante et al. 2021). The laser rapidly switches between two foci to maintain two optical traps. This reduces some sources of mechanical vibrations and drift, greatly reducing noise in the signal (Heller et al. 2014). When a flow chamber is used with multiple laminar flow channels, a dual trap can quickly be moved from channel to channel, providing near-instantaneous buffer exchange.

Typically, in dual-trap optical tweezers experiments the same, or similar, beads are used in both traps. For example, single-molecule dsDNA stretching is often performed with streptavidin-coated polystyrene beads on both traps. Instead of using the same beads in both traps, it has also been reported that beads are used with different coatings or “handles” and different sizes for the two traps and loaded via microfluidics to suit the experimental design. For instance, a study published in 2024 examined Ebola virus fusion process using two differently functionalized polystyrene beads: one bearing covalently attached biotinylated dsDNA, and the other presenting a streptavidin-biotinylated GP∆TM-membrane complex (Vaknin et al. 2024). 

The implementation of fluorescence microscopy into the optical tweezers setup has recently gained popularity (Hashemi Shabestari et al. 2017). Whereas optical tweezers provide real-time monitoring of forces and dynamics, fluorescence microscopy directly probes the localization, identity, quantity, and movement of labelled molecules. This approach can for instance be used to identify and follow labelled molecules interacting with a nucleic acid strand. Furthermore, through photobleaching assays, the signal intensity originating from a single fluorophore can be determined. This allows for quantification of the number of bound proteins. Monitoring the amount of bound proteins over time yields an adsorption curve to which adsorption models can be fitted to extract thermodynamic parameters (Marchetti et al. 2019). The fluorescence signal over time can be presented in the form of a kymograph (Marchetti et al. 2019, Buzón et al. 2021); a figure where one axis represents the spatial dimension and the other represents time. A kymograph contains information not only about the number of proteins bound but also the amount of protein that binds per binding event and the diffusion of the protein oligomers along the substrate (Marchetti et al. 2019, Buzón et al. 2021).In addition to fluorescently labelled proteins, using dyes with special properties can provide more specific information. One example is the use of intercalating dyes, a type of fluorescent dye that only fluoresces when it is intercalated between base pairs of double stranded nucleic acids (Buzón et al. 2021). This dye can thus be used to localize and monitor double-stranded regions of nucleic acid.

## Understanding viruses by using optical tweezers

### Virus particles trapped by optical tweezers

**Fig. 4:**
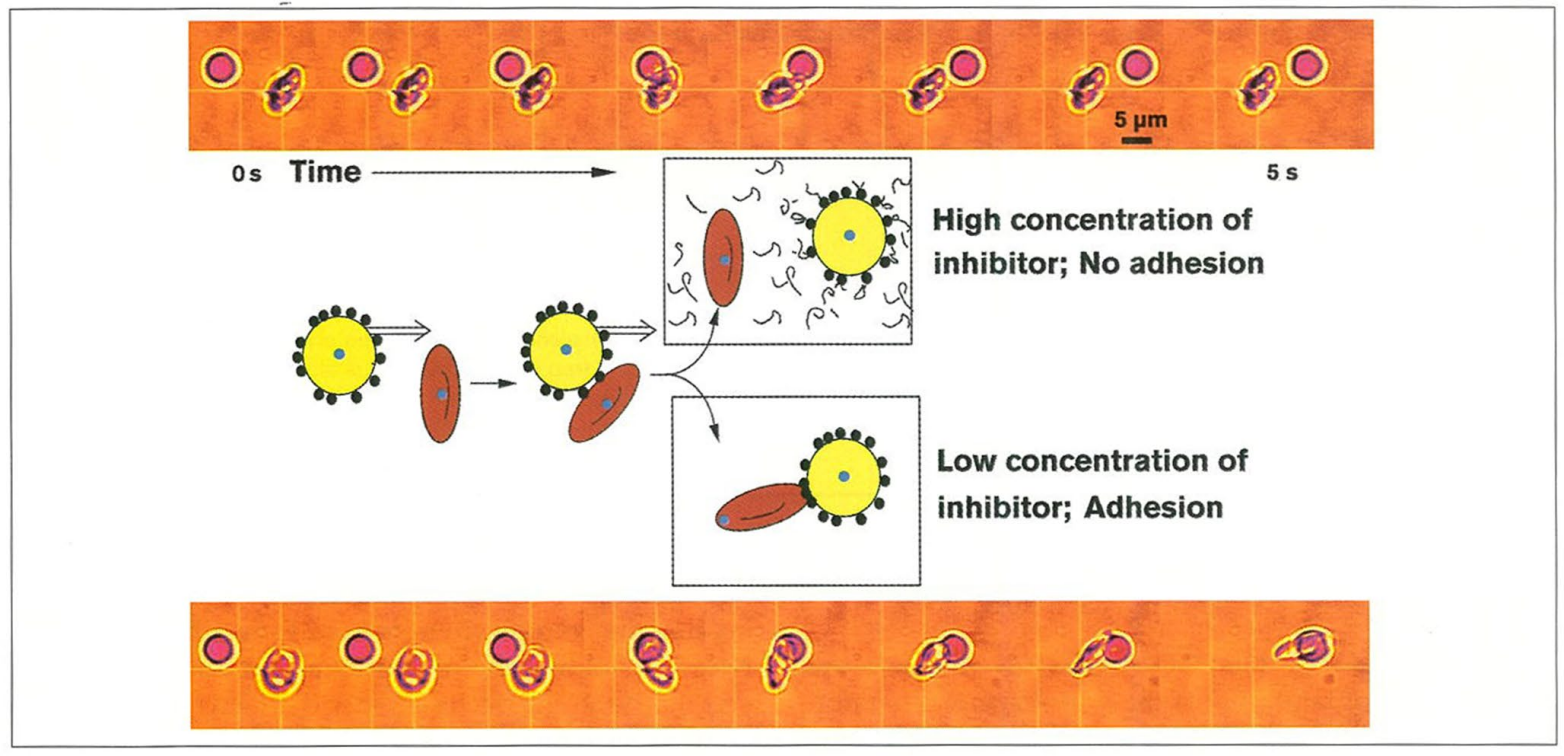
Schematic representation of an assay for controlled collision between two particles. The influenza virus-coated microsphere (yellow in the schematic) is trapped by the mobile trap and moved around. The other trap is kept stationary and traps a single erythrocyte. Reproduced from ref (Mammen et al. 1996) with permission

As early as 1987, Arthur Ashkin utilised optical trapping to manipulate viruses and bacteria. He used approximately 120 milliwatts of power of an argon laser power to trap individual tobacco mosaic viruses and groups of viruses that were closely packed together in aqueous solutions (Ashkin and Dziedzic 1987). This inspired many scientists to further investigate the use of optical tweezers for manipulating biomolecules and viruses. For example, in 1996, Mammen et al. developed a detection assay using two optical traps to study collisions between two particles under controlled biological conditions (Fig. 4). They investigated the adhesion of influenza virus-covered spheres to red blood cells during controlled collisions with various attachment inhibitors (Mammen et al. 1996). In Fig. 1 several other examples are given of the use of optical tweezers in physical virology. Ashkin’s 2000 review on the history of optical trapping and manipulation of small neutral particles, atoms, and molecules acknowledged and anticipated further research using optical tweezers to manipulate various substances such as bacteria, viruses, T cells, ribosomes and even non-biological entities (Ashkin 2000).

**Fig. 5:**
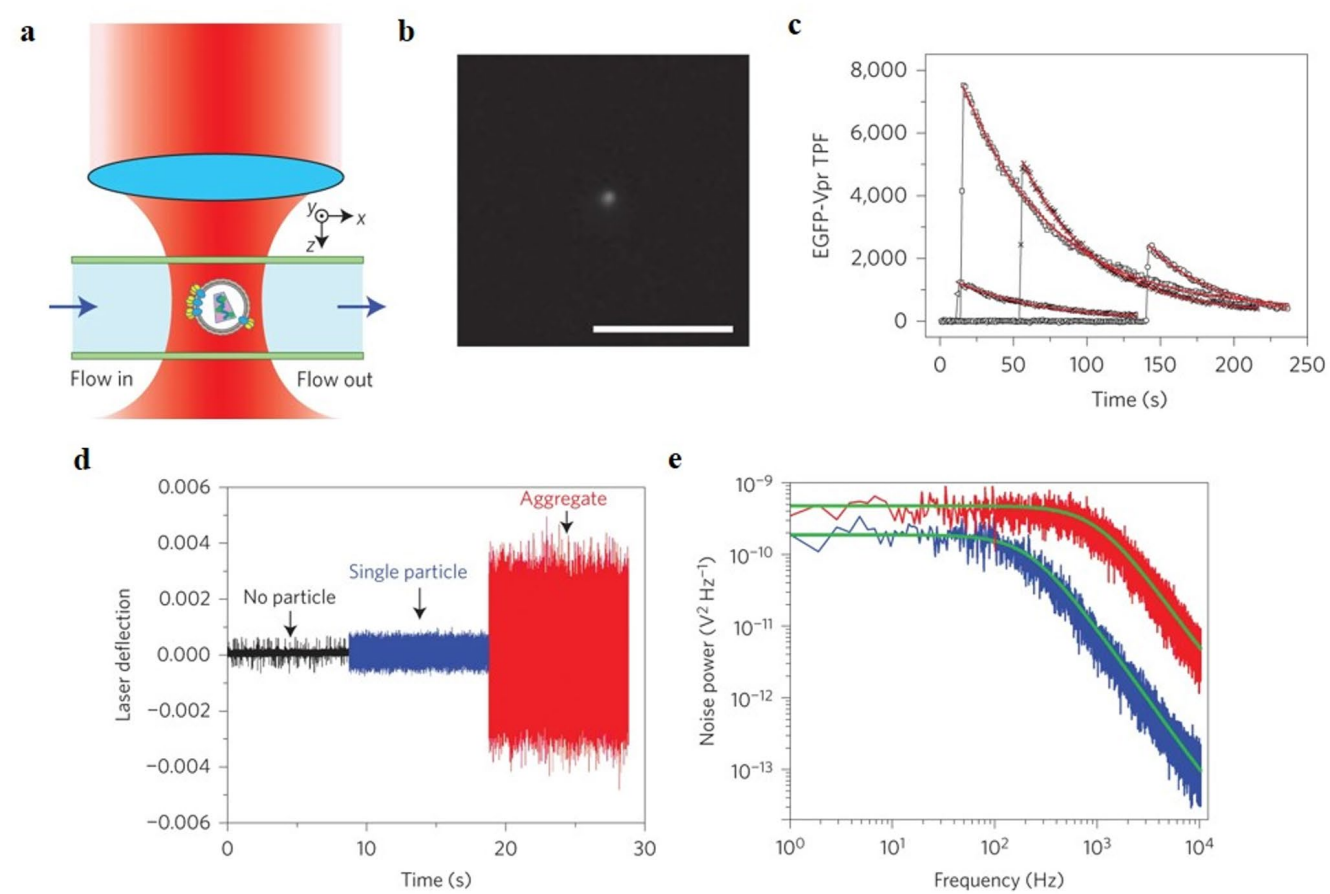
Optical trapping of HIV virions. (**a**) Schematic of the experimental setup in which individual HIV-1 virions are introduced into a microfluidic chamber and held in place using optical tweezers. Trapping is achieved with an infrared laser focused at the centre of the chamber. (**b**) Two-photon fluorescence (TPF) image of a trapped HIV virion. Scale bar, 10 μm. (**c**) Different TPF time courses from individually trapped HIV virions. (**d**) Laser deflection was measured in real time using back focal plane (BFP) interferometry. (**e**) Power spectra calculated from the data shown in d) and fits (green curves) to an aliased Lorentzian. Reproduced from ref (Pang et al. 2014)﻿ with permission

Due to the small size of viruses, which makes them prone to escaping after being trapped, and the shift in focus of optical tweezers research to DNA stretching and DNA-protein interactions, the pioneering work of using optical tweezers to manipulate individual viruses was not much followed up. However, in 2010. McNerney et al. used optical tweezers to manipulate uninfected primary CD4^+^ T cells in proximity to HIV Gag-iGFP transfected Jurkat T cells, in order to investigate the factors that induce stable adhesion (McNerney et al. 2010). After that, the Cheng lab used optical tweezers to stably trap, manipulate, and measure a single HIV-1 virion under close-to physiological conditions (Fig. 5). In this study, the live HIV-1 stock was diluted in complete media without any fixation procedures and injected into a microfluidic chamber (Pang et al. 2014). When a HIV-1 particle was trapped by the 830 nm infrared laser focused at the centre of the chamber, the virus instantly became ‘visible’ against the dark background due to the simultaneous two-photon excitation of EGFP by the trapping laser. In addition to detecting the virion by using EGFP fluorescence signals, it was confirmed that a virus was trapped by observing changes in the laser deflection at the objective’s back focal plane (BFP). Different deflection signals distinguished whether a single virus particle or the virion aggregate was trapped, allowing differentiation of virus particle sizes. Both single particles and viral aggregates exhibited behaviours consistent with the Brownian motion of a particle in a harmonic potential (Pang et al. 2014, Neuman and Block 2004).

**Fig. 6:**
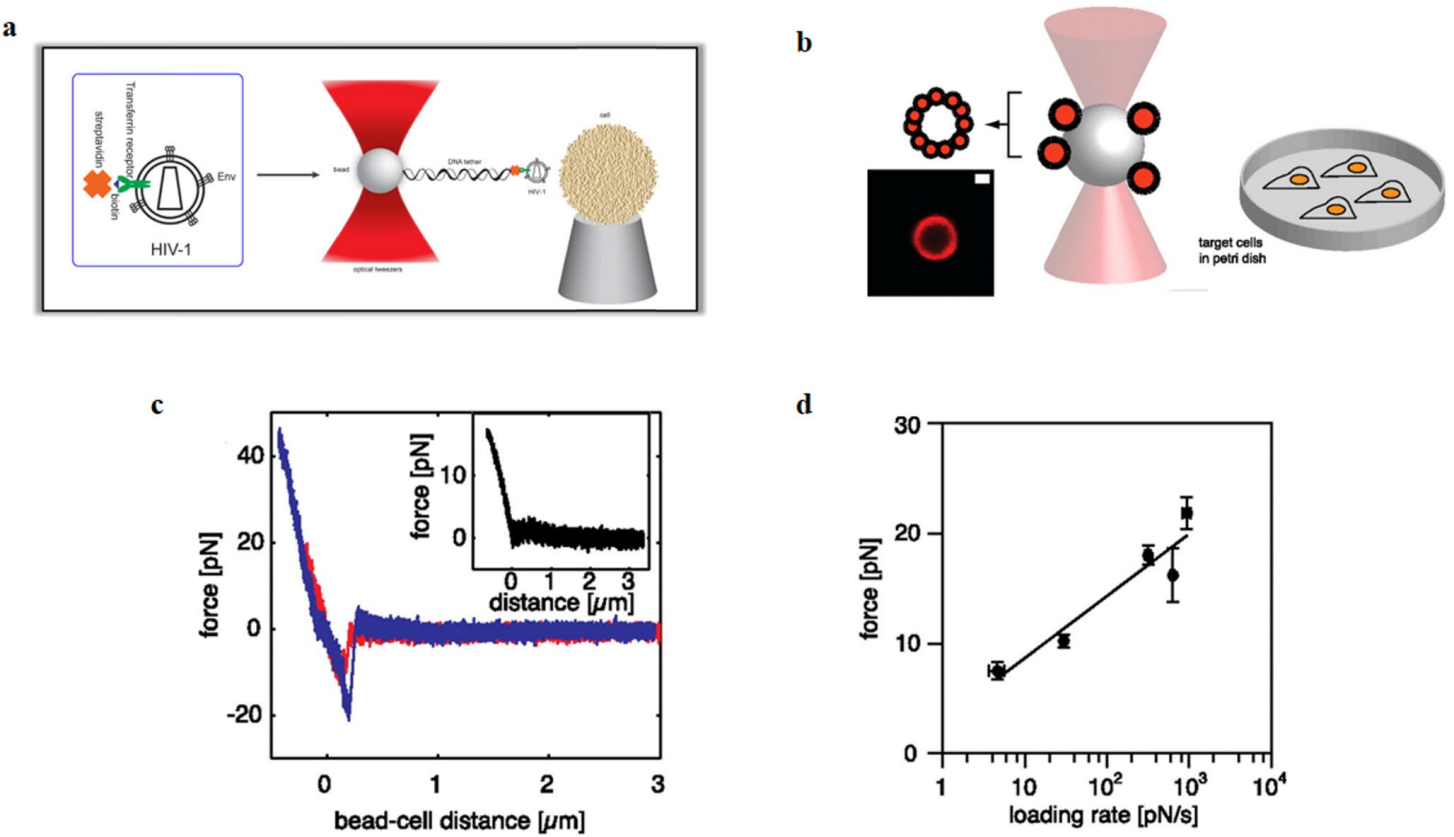
Single-virus force spectroscopy studies on virus and cell interaction. (**a**) Schematic design to tether a single HIV-1 virion at the end of a single DNA. The virion can then be manipulated through the DNA tether to probe the interaction between the virion and the cell immobilized atop of a micropipette (Schimert and Cheng 2018). (**b**) Schematic design to measure the binding forces between viruses on beads and adherent cells grown in glass-bottom Petri dishes; (**c**) Force-distance curves of virus-coated beads interacting with CHO cell, when the bead surface was blocked with BSA, no interaction could be detected (**c**, inset); (**d**) Dynamic force spectrum representing single-virus–receptor interactions on the surface of CHO cells. Each data point corresponds to a single binding event, with the most probable rupture force plotted against the loading rate. Error bars reflect uncertainties in the trap stiffness calibration and rupture force determination (Sieben et al. 2012). Reproduced from refs (Schimert and Cheng 2018, Sieben et al. 2012)﻿ with permission

In their subsequent research, the Cheng lab continued to use HIV-1 as a model virus for optical tweezers studies. They precisely determined the refractive index of individual viruses and quantified the heterogeneity between single virions with single-molecule resolution. In addition, they also developed a microtechnique to physically deliver individual HIV-1 virions into single host cells in solution (Fig. 6a), opening new avenues for studying virus-cell interactions (Hou et al. 2016, Pang et al. 2016, Schimert and Cheng 2018). Virus cell interactions have also been probed in a slightly different manner. Instead of tethering the viral particle via a DNA molecule to a bead, particles are directly coated onto beads. In this manner, the binding of influenza viral particles to cells was studied (Sieben et al. 2012). It was found that influenza A X-31 virions strongly and specifically bind to sialic acid (SA) on cell surfaces (Fig. 6b). Force measurements using virus-coated polystyrene beads revealed rupture forces of ~ 12 pN on Chinese hamster ovary (CHO) cells and up to ~ 23 pN on Madin–Darby canine kidney (MDCK) cells, likely due to higher SA density. Neuraminidase treatment significantly reduced binding probability, confirming SA-specific interactions. Dynamic force spectroscopy on CHO cells showed a linear relationship between loading rate and rupture force, indicating a single energy barrier (Fig. 6c&d). Here it can be noted that in general interpretation of loading rate-rupture force dependencies can be challenging and is simplified when interactions are rare, i.e. when on average rupture of a single bond is measured and not of multiple bonds (Merkel et al. 1999). Dengue virus (DENV) interactions with dopamine type-2 receptors (D_2_R) on cells were studied by coating polystyrene beads with DENV particles (Arifin et al. 2024). The coated beads were then brought into contact with CHO cells. The binding forces between DENV and D_2_R-expressing cells were quantified at ~ 50–60 pN, indicating specific interactions. Treatment with D_2_R antagonists significantly reduced binding, confirming the functional role of D_2_R as a DENV attachment factor. These results suggest that D_2_R is a potential receptor for DENV and may serve as a target for antiviral strategies.

**Fig. 7:**
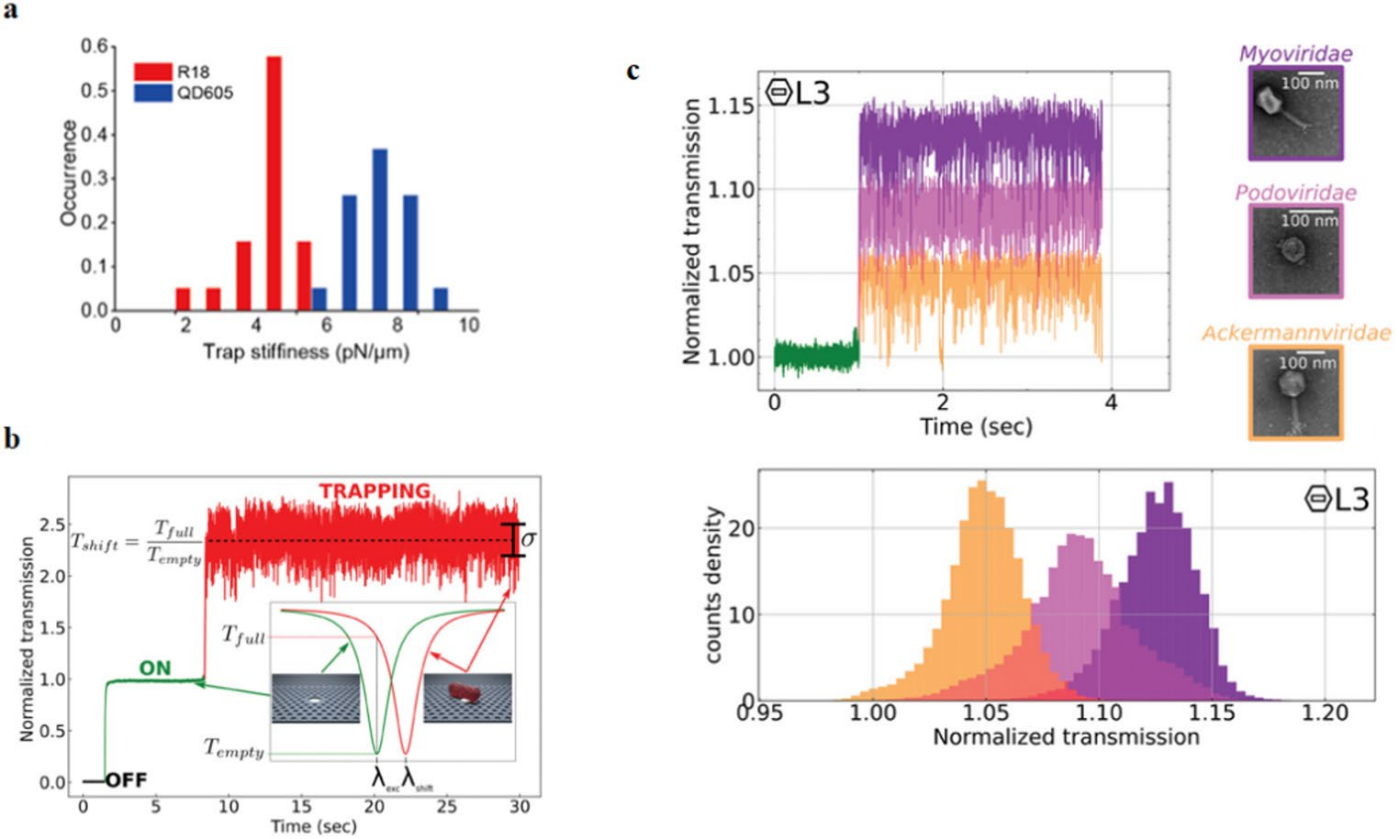
Optical trapping and detection of single viruses. (**a**) Histogram of trap stiffness for single R18- and QD-tagged H9N2 viruses (Xu et al. 2023). (**b**) Normalized transmission signal during optical trapping of a single bacterium in the photonic crystal (PhC) cavities; (**c**) Comparison of transmission properties (normalized transmission shift) upon Myoviridae, Podoviridae, and Ackermannviridae optical trapping on the L3 slot cavity (one of the PhC cavities) (Villa et al. 2024). Reproduced from refs (Xu et al. 2023, Villa et al. 2024)﻿ with permission

Recently, individual viruses were studied in an approach where simultaneously to the measurement the optical trapping of the set-up was enhanced. Using an integrated optical tweezers system with a laser confocal fluorescence imaging set-up, trapping and real-time imaging of single H9N2 viruses was performed (Xu et al. 2023). In parallel a quantitative analysis based on multiple parameters in the culture medium could be performed. It was shown that single H9N2 viruses which are labelled with quantum dots (QD) induced an increased gradient force and trap stiffness compared to unlabelled viruses (Fig. 7a). The increased trap stiffness turns out to be related to the QD-induced increase of the polarisability of the trapped construct (Xu et al. 2023). Although in this work QD-labelling was effectively used to trap viruses with optical tweezers, this method could not distinguish between multiple virus types. A study published in 2024 provided new possibilities for applying optical trapping for physical virology approaches. Unlike traditional free-space optical tweezers, which are limited by high-energy lasers and the diffraction limit of light, this paper leverages near-field optics to propose an innovative method using integrated resonant photonic crystal (PhC) cavities in silicon as optical nanotweezers (Fig. 7b&c) (Villa et al. 2024). They found that L3 slot nanocavities can distinguish between bacteria and bacteriophages without the need for labels or specific surface bio-receptors (Fig. 7c). Furthermore, adjusting the spatial extent of the resonant optical modes in a low-index medium, allowed for a precise identification of different phenotypes within a family of bacteriophages. This work offers new insights into using optical tweezers in physical virology including approaches such as phage therapy applications (Villa et al. 2024).

### Genome packaging

Viruses encapsulate the viral nucleic acid within a protein shell known as a capsid to protect their genetic material, a process referred to as genome packaging. Two distinct viral assembly pathways are observed. In one pathway, the viral genome and capsid proteins co-assemble (Buzón et al. 2020; Daudén et al. 2024; Perlmutter and Hagan 2015) while in the other, an empty capsid (procapsid) is first formed and subsequently filled with the genome by an ATP-driven molecular motor (Casjens 2011, Prevo and Earnshaw 2024). During packaging, the viral nucleic acid is condensed to very high densities. In some cryo-EM reconstructions, selected particle classes can display concentric, layered density within the capsid; however, this appearance may partly result from class averaging of subsets with higher local order, while other studies report disordered or particle-specific genome arrangements (Comolli et al. 2008, Berndsen et al. 2014, Jin et al. 2015). Thus, internal genome organization is probably not universal but system-dependent, and likely varies with capsid geometry, ionic and electrostatic conditions, and non-equilibrium packaging dynamics (Jin et al. 2015). The optical tweezers technology demonstrates unique advantages in studying virus packaging processes because it allows for the manipulation of individual nucleic acid molecules interacting with a large number of capsid proteins in a biophysical environment, thereby closely mimicking real viral packaging processes. These studies build upon previous investigations using optical tweezers to stretch single DNA or RNA molecules in both double-stranded and single-stranded forms, providing a thorough understanding of their mechanical properties (Smith et al. 1996, Bustamante et al. 2003). From the perspective of structural and physical virology, the conformational dynamics of nucleic acids play a crucial role. These stretching measurements on nucleic acids are essential for understanding the energetic barriers and the functions of proteins and molecular complexes involved in nucleic acid packaging. In this section we will first focus on studies of the packaging of the viral genome into a preformed capsid by an ATP-driven molecular motor. Next we will discuss self-assembly around the genome.

#### Genome packaging using molecular motors

**Fig. 8:**
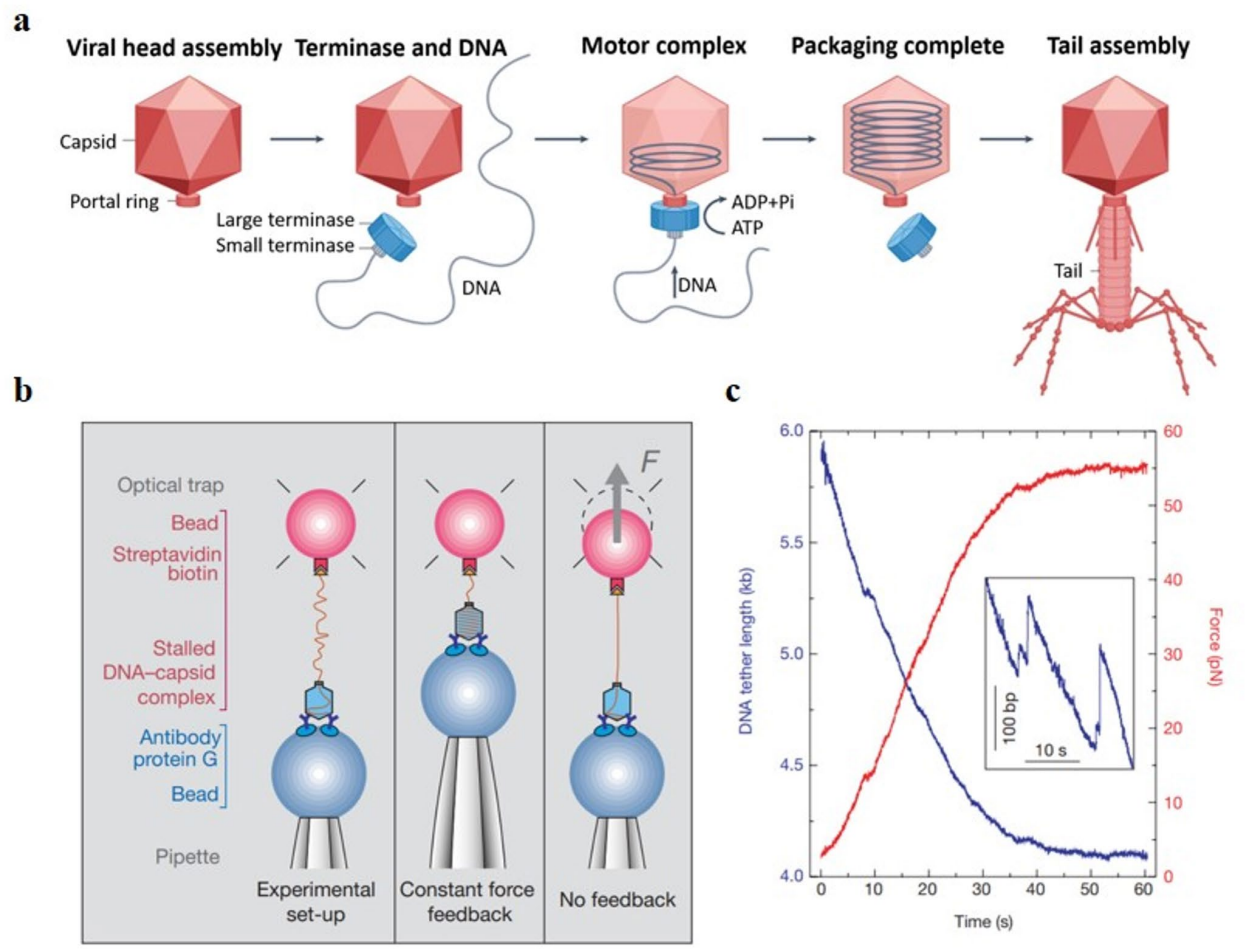
Studying bacteriophage genome packaging with optical tweezers. (**a**) Cartoon showing the assembly process of a bacteriophage (Prevo and Earnshaw 2024). (**b**) Experimental setup (Smith et al. 2001). (**c**) Characteristic traces showing the packaging of DNA in constant force mode (blue) and the large force generation by the$\:\:{\upphi\:}$29 packaging motor in constant extension mode (red), stalling around 55 pN. Inset: zoom showing random slipping events (Smith et al. 2001). Reproduced from refs (Smith et al. 2001, Prevo and Earnshaw 2024)﻿ with permission

Many tailed double stranded DNA bacteriophages, but also for instance Herpes Simplex Virus first assemble a genome-less capsid, the so-called procapsid or prohead. The procapsid assembles with the aid of scaffolding proteins, and once the shell closes the scaffold is enzymatically cleaved and exits through small holes in the shell. The place where the scaffold used to be, can now be taken up by the viral genome. For the dsDNA phages like phi29, lambda, and T4, but also other viruses, there is a portal protein (head-tail connector protein) that is involved in capsid assembly and forms a multisubunit ring that forms a portal channel in the fully assembled capsid. The packaging motor docks onto this ring and the DNA both enters and exits the capsid through this portal structure, which is located at a unique vertex of the capsid (Fig. 8a). The packaging machinery typically comprises the portal and a terminase complex that binds to it. The terminase contains a large terminase subunit, the packaging ATPase and a small terminase subunit, the DNA recognition protein, with oligomeric states and stoichiometries that vary across phage systems (Casjens 2011) The motor is located at one of the vertices of the empty procapsid (Sun et al. 2010). The packaging motors are able to package several micrometers-long molecules of DNA into procapsids with only 20–100 nm in diameter. This process requires the motor to overcome huge energetic and entropic penalties and resist significant pressures from inside the capsid (Sun et al. 2010). Early single-molecule studies around 1995 developed optical-tweezers methods to manipulate individual DNA molecules and applied them to quantify fundamental physical properties such as relaxation dynamics and elastic responses (Perkins et al. 1994, Smith et al. 1996). By innovatively labelling the DNA ends with biotin, the manipulation of nucleic acids became easier. These early approaches were subsequently extended from bare nucleic acids to viral systems, enabling direct measurements of the mechanics and dynamics of packaging DNA into capsids. The dsDNA bacteriophages $\:{\upphi\:}$29,$\:\:{\uplambda\:}$, and T4 are the most extensively studied phages using optical tweezers. In a pioneering study by the Bustamante lab in 2001 packaging of $\:{\upphi\:}$29 dsDNA was followed for the first time at the single molecule level (Smith et al. 2001).

To measure DNA packaging by the motor, they first initiated packaging and then halted it with the non-hydrolyzable ATP analog ATP- γS. The entire complex was tethered at the unpackaged DNA end to a streptavidin-coated microsphere, which was injected into a microfluidic chamber containing ATP. This bead was caught in an optical trap. A second microsphere coated with anti-$\:{\upphi\:}$29 antibodies was introduced and captured with a micropipette (Fig. 8b&c). By controlling and moving the bead’s position, the two microspheres were brought close enough for the prohead to bind with the anti-$\:{\upphi\:}$29 microsphere within seconds, forming a stable tether between the two beads (Smith et al. 2001). These experiments revealed that the $\:{\upphi\:}$29 motor exerts a force of up to approximately 60 pN on the DNA, establishing it as one of the most powerful biological motors known. Under conditions of saturating ATP, the maximum packaging rate was around 100 bp/s. However, once the prohead was more than 50% filled, the packaging rate began to decline, approaching zero as the viral genome, about 6 μm in length, neared complete packaging. By comparing these data with the force-velocity relationship measured under external load, the study suggested that an electrostatic counter-force builds up within the capsid during packaging. To further investigate this hypothesis, Fuller et al. systematically examined the effects of ionic conditions on DNA packaging (Fuller et al. 2007). Their results showed that ionic strength independently modulates both motor activity and the internal forces resisting DNA confinement. While Mg^2+^ (~ 1 mM) is essential for motor function, the addition of 100 mM Na^+^ significantly increases the motor’s velocity in the absence of load. In contrast, internal forces resisting packaging were found to be up to ~ 80% higher when Na⁺ was the dominant ion compared to Mg^2+^. Additionally, increasing the total ionic strength reduced the internal force, supporting the idea that electrostatic repulsion is a key contributor to the internal resistance encountered during packaging.

A few years later, Smith’s group improved the system by attaching the pre-assembled head-motor complex to one microsphere and the DNA to a second microsphere (Rickgauer et al. 2008). When brought close together, the motor binds to the DNA and initiates packaging. This setup allows for the measurement of DNA translocation from start to finish, enabling the study of previously uncharacterized early stages of packaging and facilitating more accurate measurements of the DNA length being packaged. The experimental results could be extrapolated to an internal force around 110 pN, under the assumption that motor velocity depends only on opposing load. However, subsequent studies showed that only part of the slowing of the motor with capsid filling was due to internal force, while a significant fraction arises from allosteric regulation of the ATP-binding rate as DNA density increases, revising the internal-force estimate to ~ 20–30 pN (Berndsen et al. 2015; Liu et al. 2014). It was found that the DNA translocation rate significantly declined during the first half of head filling, rather than remaining constant. Additionally, the average DNA translocation rate at the start of packaging was approximately 165 bp/s, which is about 50% higher than the previously measured average rate of ~ 100 bp/s under 5 pN force-clamp conditions with partially filled capsids (Smith et al. 2001). The authors attribute this difference to two main factors: the speed at near-zero fill is approximately 25% higher than at one-third fill, and the speed at near-zero load is approximately 15% higher than at 5 pN. The remaining ~ 10% difference is attributed to the new initiation method used in this study and the more accurate determination of fill levels enabled by the dual optical tweezers instrument.

**Fig. 9:**
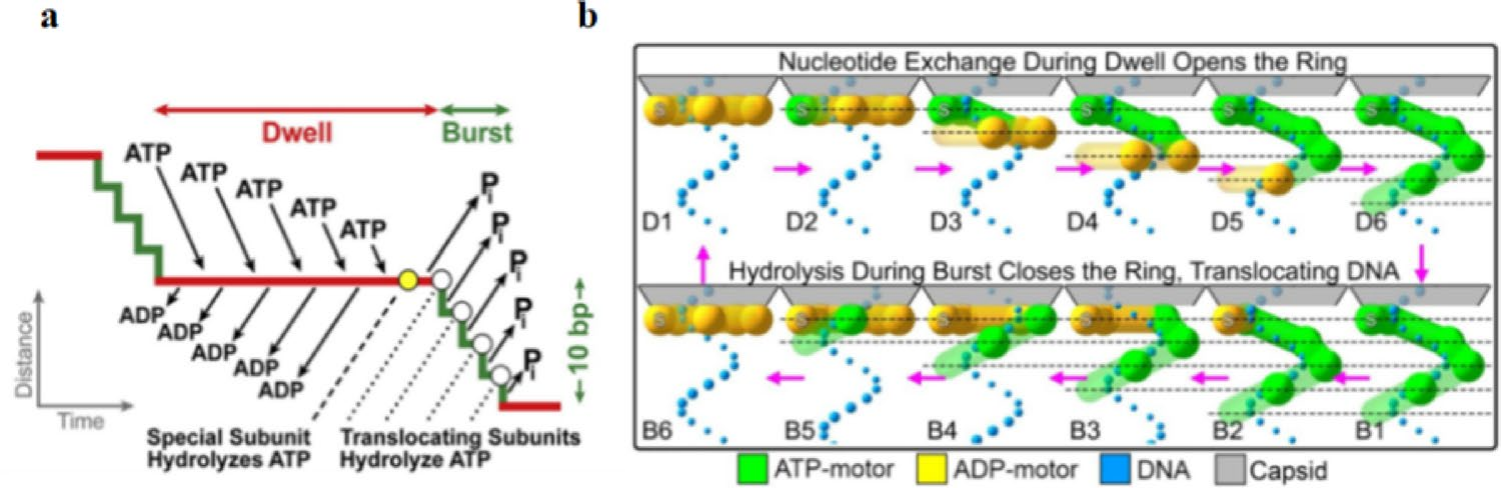
Summary schemes of the mechanochemical cycle that Bustamante’s group has established over decades of work on the $\:\phi\:$29 DNA packaging motor. **a**) Schematic showing the chemical processes taking place during the dwell and burst cycles (Liu et al. 2014). **b**) Schematic explanation of the helical inchworm mechanism (Tong and Bustamante 2021).Reproduced with permission from refs (Liu et al. 2014, Tong and Bustamante 2021)

Additionally, Bustamante has been consistently curious about the dynamic working modes of the $\:{\upphi\:}$29 DNA packaging motor (Bustamante and Yan 2022). His lab has scrutinised the effects of various experimental parameters, such as ATP concentration, structural changes in the DNA substrate, and mutations in the motor proteins, on the stepping behaviour of the DNA packaging motor. The initial motor velocity in saturating ATP (0.5 mM) decreases with increasing force (Chemla et al. 2005). Using high-resolution optical tweezers, they discovered that packaging proceeds in 10 base pair (bp) increments. Statistical analysis of the dwell times preceding these increments indicates that multiple ATP molecules bind during each dwell phase. When high force is applied, it becomes evident that each 10 bp increment is actually composed of four 2.5 bp steps (Moffitt et al. 2009). Around the same time, they aimed to determine what interactions between the motor and DNA were responsible for the observed stall forces up to 70 pN. They created DNA molecules with varying segment sizes (5, 9, 10, 11, 15, and 30 bp) where the phosphates had been neutralized. By neutralizing specific strands, they demonstrated that the motor contacts two adjacent DNA phosphates every ten base pairs during the dwell phase, but only in the strand packaged in the 5’ to 3’ direction (Aathavan et al. 2009). After that, they investigated the coordination between motor subunits in the DNA packaging mechanism. Using ATP-γS, a non-hydrolyzable ATP analog, it was discovered that binding of a single analog induced pausing in the motor, revealing that the motor subunits retain coordination during these pauses and produce bursts of 10 bp increments. Further, Na^+^-orthovanadate was used to show that preventing ADP release also caused pausing, indicating a coordinated ADP release during the dwell phase. This explained the motor’s apparent lack of cooperativity. Additionally, it was found that the non-mechanical subunit also binds and hydrolyzes ATP, resolving discrepancies between bulk and single-molecule studies, showing that five ATPs are consumed per cycle, with only four contributing to DNA movement (Chistol et al. 2012). Next, the role of the special subunit in the motor under normal conditions was addressed. It was demonstrated that the $\:{\upphi\:}$29 motor not only generates force but also introduces torque into the DNA during packaging. To ensure this observation wasn’t due to DNA coiling inside the capsid, the viral heads were subjected to repeated freezing and thawing to open holes so that DNA could leak out. Under these conditions, the motor still rotated the DNA at 1.5 ± 0.2 degrees per base pair. It was also observed that as the capsid filled, the burst size decreased from 10 bp to 9 bp, likely due to increased pressure. These changes suggested that the special subunit contacts the DNA phosphate at the end of each 10 bp burst, regulating the motor cycle. The motor actively rotates the dsDNA substrate by 14 degrees per burst to maintain this contact. The rotation compensates for the decreasing burst size, allowing the special subunit to maintain contact with the dsDNA and regulate the motor’s mechanochemical cycle (Liu et al. 2014)(Fig. 9). Next, mutants were created that could disrupt this coordination, specifically targeting the arginine residue R146, and it was observed that motors with a single mutant subunit behaved similarly to motors with one ATPγS bound. However, in this case, the altered phenotype was permanent and inherent to the mutant motor (Tafoya et al. 2018).

In a recent study, the team conducted a dynamic analysis of the packaging steps involving various nucleic acids. The packaging motor undergoes a cyclic process, alternating between an ATP loading dwell and a hydrolysis burst, which enables it to package one turn of DNA in four distinct steps. A helical inchworm translocation mechanism was proposed, suggesting that during the ATP loading dwell, the motor adopts a lock-washer structure, which transitions back to a planar form with each power stroke during the burst. This mechanism elucidates the motor’s differential behaviour when encountering different nucleic acids. Specifically, when faced with DNA-RNA hybrids and dsRNA, the motor adjusts its burst to accommodate the shorter helical pitches by shortening the fourth step while maintaining consistency in the first three steps. Additionally, intermittent loss of grip on RNA-containing substrates suggests that the motor optimally engages with dsDNA for load-bearing interactions (Castillo et al. 2021).

In addition to the work on$\:\:{\upphi\:}$29 by the Smith and Bustamante labs, the mechanochemical properties of other viral packaging motors have been studied as well. The Smith lab expanded the application of optical tweezers to study bacteriophage λ and the Smith and Chemla labs did the same for phage T4, using – in both cases - assays similar to those developed for$\:\:{\upphi\:}$29. Their research revealed that high force generation is a common characteristic of viral packaging motors although their packaging approaches differ. Notably, they discovered that the T4 motor operates at an exceptionally high translocation rate, averaging 700 bp/s and reaching maximum rates of up to 2,000 bp/s (Fuller et al. 2007). For λ phage, their studies detected procapsid expansion occurring at 30% of the total packaging process (Fuller et al. 2007). Building on these systems, single molecule optical tweezers measurements with mutant lambda and T4 motors mapped residue level determinants of performance, identifying the Q motif that links ATP recognition to DNA engagement, a conserved helix loop region that tunes translocation velocity and processivity, and core P loop NTPase elements including Walker A and Walker B that coordinate ATP hydrolysis with mechanical output (Tsay et al. 2009, Tsay et al. 2010, delToro et al. 2016, delToro et al. 2019, Ortiz et al. 2019). Additional work revealed that a catalytic glutamate acts with an arginine toggle to mediate hydrolysis and mechanochemical coupling, and supported an electrostatic contribution to force generation in T4 by showing that changes to charged residues and ionic conditions modulate stall forces and velocity (Migliori et al. 2014). Together these results establish that specific residues and motifs set ATP binding and hydrolysis rates, govern coupling efficiency, and tune both velocity and maximal force across viral packaging motors.

Subsequently, Rao and colleagues reported similar packaging activity rates for both prohead and previously filled heads of T4 bacteriophage. They discovered that fully packaged heads, as well as those emptied of most of their DNA, can reassemble and reuse the packaging machinery. Additionally, they found that various exogenous DNA molecules can serve as substrates to fill the T4 capsid through multiple initiation steps (Zhang et al. 2011). In 2017, they also investigated the package motor’s operating speed and found that the mutated motor’s ATP hydrolysis rate decreased around 2.5 times, and the DNA packaging speed decreased by 2.5 to 4.5 times. Additionally, the activation of protein gp16 was found to be required to rapidly stimulate the ATPases (Lin et al. 2017).

In 2018, collaborative work by the Smith and Rao groups on the T4 packaging motor showed nucleotide dependent DNA gripping and identified a DNA end clamp mechanism that limits genome exit once packaging has begun, establishing how gripping states and channel architecture regulate processivity under load (Ordyan et al. 2018). In 2023, the Smith group extended these measurements to the lambda motor and quantified both nucleotide independent gripping and nucleotide dependent gripping and friction during translocation. Comparison of T4 terminase complexes that lack TerS with lambda terminase complexes that include TerS suggested that TerS is a key determinant of processivity and functions as a sliding clamp that limits backward slippage when TerL transiently loses grip (Rawson et al. 2023). Together these results show that both T4 and lambda packaging complexes use a “DNA end-clamp” mechanism and that nucleotide controlled gripping modulates processivity during genome packaging.

To conclude on optical tweezers studies on dsDNA packaging of tailed bacteriophages we can summarise briefly. The characterisation of the $\:{\upphi\:}$29, λ and T4 packaging motors on the basis of their force, speed, and processivity yields the following. Packaging speeds vary significantly among different phages. The slowest is observed in $\:{\upphi\:}$29, with a rate of approximately 100 bp/s (Smith et al. 2001), while phage λ shows an intermediate speed of around 600 bp/s (Fuller et al. 2007). Phage T4 exhibits the highest packaging rate, averaging about 900 bp/s (Fuller et al. 2007; Kottadiel et al. 2012), and in some instances reaching up to 2000 bp/s (Fuller et al. 2007).

The genome length of the bacteriophages follows a similar trend, indicating that there exists a relationship between genome size and packaging speed, as the different phages have similar infectious lifetimes, all packaging their genome in ∼3–5 min (Rao and Feiss 2015). A general observation in all these packaging studies is that motor velocity decreases with increasing capsid filling and, therefore, with increasing resisting force (Smith et al. 2001, Fuller et al. 2007, Fuller et al. 2007, Fuller et al. 2007). These packaging motors are highly processive; they are capable of translocating long segments of DNA (several kbp) only in bursts of 10 bp, thus completing many catalytic cycles at a time. Pausing and slipping of the motors were observed as random processes in all these motors. The packaging motors are estimated to have an energy efficiency of at least 30% (Rao and Feiss 2015), based on the free energy change of ATP hydrolysis (∼120 pN∙nm), an experimentally determined lower bound of the maximum force of ∼60 pN, and a ∼2.5 bp step size per ATP hydrolysis. The ability of bacteriophage packaging motors to overcome the large energetic penalty of DNA packaging while tightly coordinating five ATPase subunits offers valuable insights into the biology of bacteriophages as well as the chemistry and physics of strong molecular motors.

However, molecular motor-based genome packaging does not only occur in DNA viruses. In 2017, Hanhijärvi et al. reported the pioneering use of optical tweezers to explore the details of RNA packaging in bacteriophage$\:\:{\upphi\:}$6 (Hanhijärvi et al. 2017). The complexity of this packaging process is attributed to the virus’s genome. Bacteriophage$\:\:{\upphi\:}$6 is a dsRNA virus with a tripartite genome, where the positive (+) single-stranded RNA (ssRNA) genome precursor is enclosed within a pre-formed procapsid (PC), facilitating the synthesis of the negative strand. The findings revealed that the packaging process occurs intermittently, alternating between slow and fast phases, which likely reflects differences in the unfolding of RNA secondary structures. Despite an overall low average packaging speed (0.07–0.54 nm/s), the speed can peak at 4.62 nm/s during rapid packaging phases.

#### Assembly around the genome

**Fig. 10:**
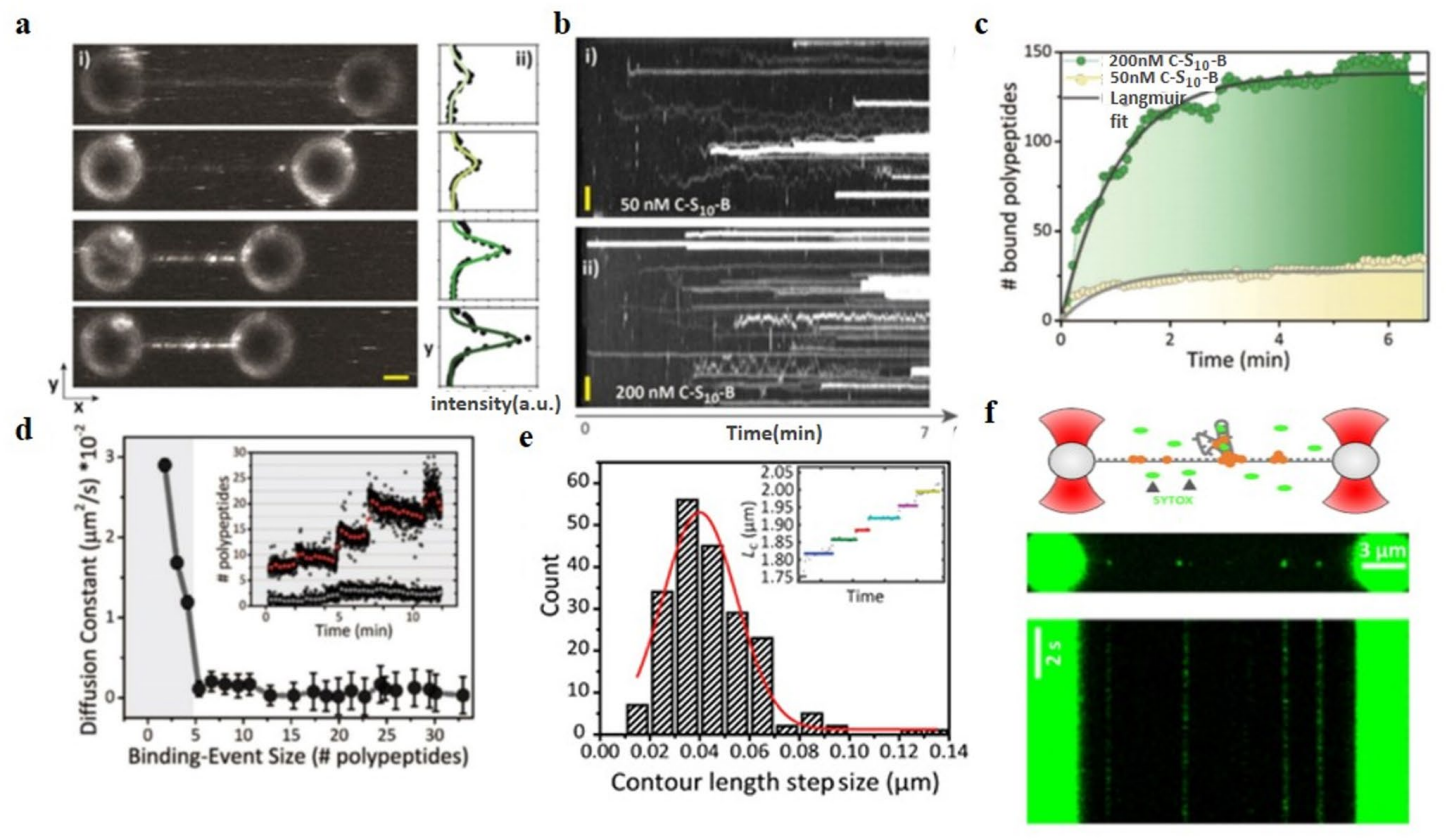
Assembly around the genome (Marchetti et al. 2019; Rosmalen et al. 2020; Buzón et al. 2021). (**a**) Progressive DNA packaging by capsid-like polypeptides (i) is accompanied by an increase in fluorescence intensity (ii), directly correlating polypeptide binding with DNA packaging. (**b**) Kymographs showing polypeptide binding onto DNA over time for two different concentrations. **c**) Binding of polypeptides over time from the kymograph in **b**), showing that polypeptide binding follows Langmuir adsorption behaviour. **d**) Diffusion constant of polypeptide oligomers, showing a sharp drop in mobility for oligomers with more than 5 polypeptides. Inset: single oligomer sizes over time, showing that growth is much more likely when starting off with an oligomer with more than 5 polypeptides. **e**)The rupture step size of SV40 VP1 pentamers distribution, average step size of 40 ± 1 nm. **f**) Schematic of a setup in which the intercalating dye SYTOX green is used to localize HBV capsid protein-induces dsDNA regions on the substrate, image and kymograph showing that dsDNA regions are stable over time. Reproduced with permission from ref. (Marchetti et al. 2019; Rosmalen et al. 2020; Buzón et al. 2021)

Most single-stranded genome viruses assemble via a passive co-assembly pathway, where the genome is compacted by the capsid protein simultaneously with capsid assembly. It has been observed for many different viruses that infectious virions spontaneously form in buffers containing only capsid proteins and genetic material, thus showing that this is a passive pathway, independent of enzymatic activity or other energy sources. Thus, assembly tends towards a minimum in the energy landscape while significantly compacting long nucleic acid molecules (Buzón et al. 2020). The genome acts as a template around which the capsid protein assembles and an example of virus-like protein induced shortening of a nucleic acid strand is shown in Fig. 10.

**Fig. 11:**
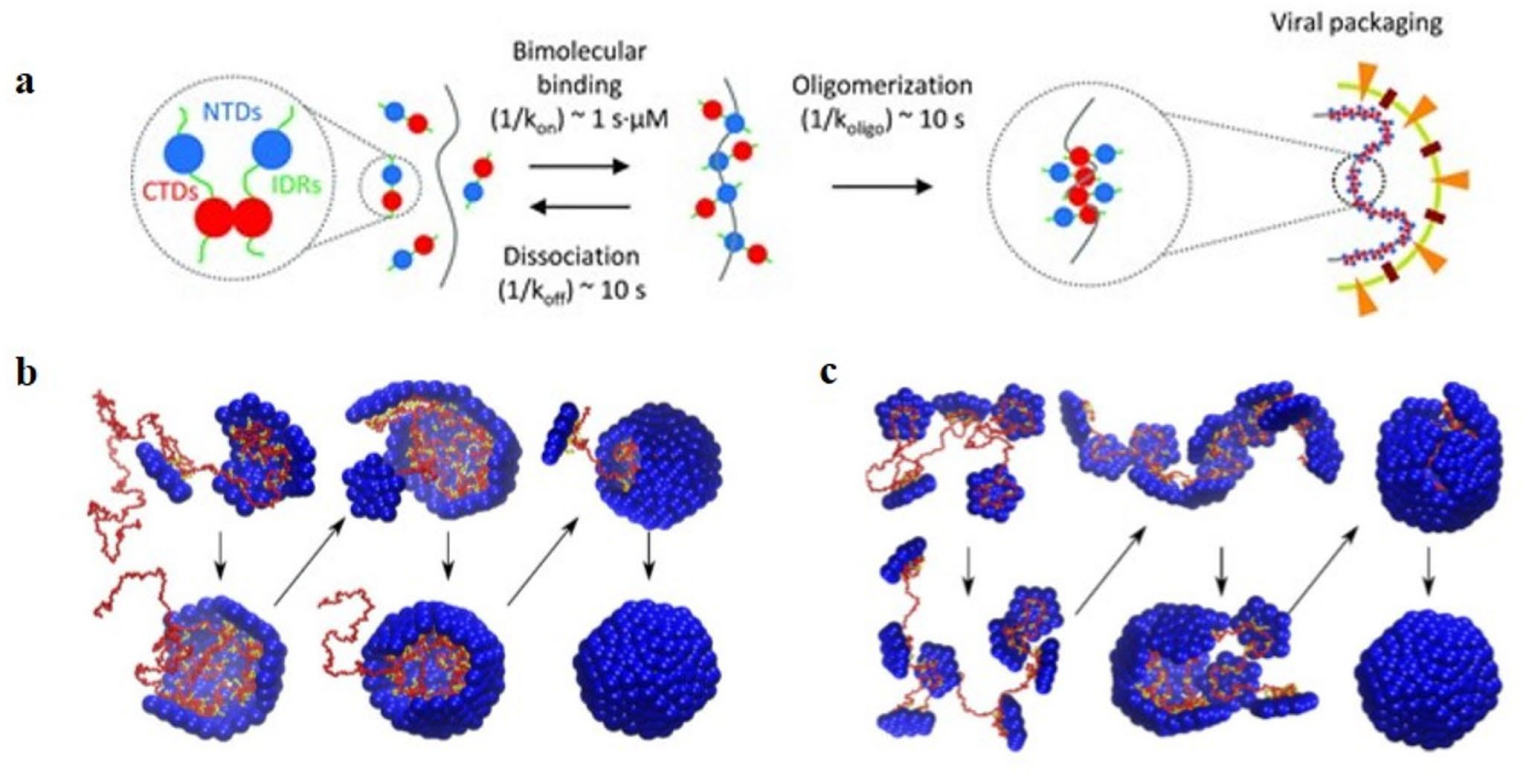
Characterization of the viral packaging. (**a**) Model of multistep process of N protein binding and packaging vRNA (Morse et al. 2023). (**b**) Ordered nucleation-and-growth pathway dominated by strong protein-protein interactions (Perlmutter et al. 2014). (**c**) Disordered en-masse pathway dominated by protein-substrate interactions (Perlmutter et al. 2014). Reproduced from refs (Morse et al. 2023, Perlmutter et al. 2014)﻿ with permission

Based on theory and computer simulations, various pathways for capsid assembly have been formulated, dependent on the balance between protein-protein and protein-genome interactions (Perlmutter et al. 2014, Zlotnick et al. 2013). Two of these are an ordered nucleation-and-growth mechanism with strong protein-protein interactions (Fig. 11b), and a disordered en-masse pathway with weaker protein-protein contacts (Fig. 11c). However, the kinetics and dynamics are much more difficult to study in situ due to the heterogeneity of the system. Only relatively recently it has become possible to study viral assembly processes with real-time single-particle methods (Marchetti et al. 2019). These methods, including optical tweezers, are a powerful tool in elucidating this process as they give real-time insights into single particles, as well as ensemble averages and distributions. Assembly can be studied in quasi-native environments, and factors such as salt concentration and pH are easily tuned. Most single particle studies combine several different techniques, all providing complementary information, to construct a view of the dynamics of assembly processes as complete as possible. In this section, some key contributions of optical tweezers experiments to this field are highlighted.

In one of the first single-particle studies on helical VLP assembly (Marchetti et al. 2019), a dsDNA molecule was tethered between two beads and then exposed to a synthetic polypeptide to study the dynamic process of peptide assembly into rod-shaped virus-like particles. This system served as a model for Tobacco Mosaic Virus (TMV) and the polypeptides were fluorescently labelled. Introducing fluorescence methods to an OT experiment is a very powerful method to increase the amount of information that can be obtained. The experimental results showed the movement of fluorescent particles of different sizes on the DNA substrate. Furthermore, it was shown that the DNA tether is shortened as fluorescence intensity increases (Fig. 10a), and thus as the number of bound polypeptides increases. Following the fluorescence intensity over time showed that the binding kinetics agree well with the relatively simple Langmuir adsorption model (Fig. 10c), allowing calculations of equilibrium constants and binding free energy changes. Additionally, the absolute number of polypeptides per binding event could be characterized. Monitoring the mobility of polypeptide oligomers along the genome revealed that for oligomers larger than five subunits, the diffusion constant dropped to essentially zero (Fig. 10d). This suggests a classical, ordered nucleation-and-growth-like mechanism (Marchetti et al. 2019) with strong protein-protein interactions (Fig. 11b), where the critical nucleus size is five proteins.

Besides helical viruses, this approach has also been employed in studying assembly processes of icosahedral capsids. Simian Virus 40 (SV40) virus-like particles were used as a model system to reveal the multi-step assembly mechanism of SV40 VLPs around dsDNA (Rosmalen et al. 2020). Initially, the binding of VP1 pentamers to DNA resulted in a significant reduction in persistence length. Additionally, the pentamers appeared to stabilize DNA loops. Subsequently, interactions between pentamers led to intermediate structures with reduced contour lengths. These structures then stabilized into particles that permanently shortened the contour length to a degree consistent with the DNA compression observed in wild-type SV40. This data suggests that a multi-step mechanism can lead to the fully assembled cross-linked SV40 particles. In particular, this pathway involves strong protein-genome interactions and weaker protein-protein interactions, giving rise to a disordered pathway (Fig. 11c). VP1 pentamers rapidly bind to DNA, thereby increasing the local concentration of VP1 pentamers with DNA acting as an antenna, then multiple DNA binding sites between each VP1 pentamer form loops, leading to further DNA compression. Additionally, this compression will form many intermediate structures by increasing the local concentration of pentamers, and then the intermediate structures will stabilize over time due to the formation of disulfide bonds.

In 2021, features of the packaging process of HBV virus were studied by focusing on the capsid-protein genome interactions. This was innovatively performed by combining fluorescence optical tweezers and high-speed atomic force microscopy to further enhance the resolution. The changes in the apparent single-stranded DNA (ssDNA) contour length under HBV capsid protein (Cp) assembly conditions was reported. The results indicated packaging of approximately 1000 bases within about 3 min, suggesting active packaging by Cp dimers. Also, most traces showed packaging occurring through continuous assembly and disassembly steps, highlighting the reversibility of the process. They extracted step sizes from the packaging trajectories, revealing two populations of step sizes, one approximately − 70 bases (assembly) and another approximately + 64 bases (disassembly) (Buzón et al. 2021). This suggests that Cp exhibits an optimal assembly configuration, or assembly footprint, resulting in the packaging of ~ 70 nucleotides (nt). These experiments indicate that Cp is capable of condensing nucleic acids even when the genome is under tension. Additionally, through fluorescence microscopy results, it was found that Cp-nucleic acid interactions promote DNA compaction (Buzón et al. 2021). 

This research extended to studying the structural assembly processes of various coat proteins, allowing to compare the energy involved in self-assembly initiation across different viruses. Specific, contact-rich pentameric arrangements of HBV coat proteins were identified as a critical intermediate in the assembly pathway. This work also elucidated the energy balance required for the self-assembly process, providing deeper insights into the factors that drive and stabilize viral assembly.

The dynamics of protein-genome interactions can especially well be probed with an OT force spectroscopy experiment. This has for instance been done for SV40 (Rosmalen et al. 2020), hepatitis B virus (HBV) (Buzón et al. 2021) and human immunodeficiency virus-1 nucleocapsid protein (HIV-1 NC) (Williams et al. 2001, McCauley et al. 2015, McCauley et al. 2020). In such an experiment, the DNA molecule is subsequently stretched and relaxed multiple times. Several models for polymer behaviour exist, such as the worm-like-chain (WLC) model (Smith et al. 1996, Bouchiat et al. 1999, Kricheldorft et al. 1995) or the extensible freely jointed-chain (eFJC) model (Smith et al. 1996, Bustamante et al. 1994). By fitting polymer models to the force-extension curves, several physical parameters can be extracted. An important parameter is the persistence length, a measure in polymer physics that determines the maximum length over which the direction of the contour of the polymer remains correlated, i.e. it is a measure of polymer stiffness. For HBV, it was shown that persistence length increased with Cp concentration in a saturable manner (Buzón et al. 2021). From these concentration-dependent dynamics experiments, the energetics of the Cp-DNA interaction could be characterized by fitting the MGvH model. Combining the calculated energetics with high speed AFM (HS-AFM) measurements, which revealed a pentamer of dimers as an important intermediate structure, suggested that the work per condensation step (~ 100 *k*_*B*_*T*) corresponds well with the free energy change associated with formation of a pentamer on ssDNA ($\:\varDelta\:$*G* = 90 ± 10 *k*_*B*_*T* ) suggesting that a single pentamer of Cp dimers could facilitate DNA compaction and serve as a nucleus for capsid growth (Buzón et al. 2021).

An intriguing example of a viral genome packaging mechanism that does not directly participate in the initial self-assembly phase of the viral life cycle is the activity of the HIV-1 nucleocapsid (NC) protein during and after reverse transcription. HIV-1 is an enveloped retrovirus whose genome is enclosed within a capsid composed of the capsid protein; however, genome compaction is mediated by NC protein. During infection, the vRNA genome is reverse transcribed into dsDNA, which is integrated into the host genome after capsid uncoating. Recently, it was shown that capsid uncoating requires the pressure build-up of full-length dsDNA and that premature uncoating causes the DNA to be degraded by the host, thus indicating that successful infection requires reverse transcription to take place entirely within the tiny free volume of the capsid (Burdick et al. 2024). During reverse transcription, NC is known to function as a nucleic acid chaperone, facilitating the destabilization of secondary RNA structures, which has also been studied using optical tweezers (Williams et al. 2001, McCauley et al. 2015, McCauley et al. 2020, Post et al. 2016). Aside from acting as a chaperone for reverse transcription, it has been found that NC is important in compacting the newly synthesized DNA (Gien et al. 2022, Gien et al. 2024). Notably, NC is believed to play a critical role in regulating capsid uncoating in response to reverse transcription (Gien et al. 2022, Gien et al. 2024, Müller et al. 2022), by condensing the newly synthesized dsDNA, keeping the capsid intact until after reverse transcription is complete. The role of NC in compacting DNA and thus protecting the viral genome until reverse transcription is complete, highlights its relevance to the field of genome packaging, as it effectively organizes nucleic acids similar to self-assembly. Compaction of dsDNA by NC was studied with fluorescence-optical tweezers and atomic force microscopy (Gien et al. 2022, Gien et al. 2024), where it was shown that NC alone could fully compact an extended dsDNA molecule into a globular particle. Consistent with the AFM measurements, it was observed that a globule of fluorescently labelled NC forms on an unstretched dsDNA molecule, which decreased in intensity when tension was increased. In the force-extension measurement (Gien et al. 2022), the DNA-NC interaction was separated into two steps; protein-free DNA was first stretched with forces up to 20 pN, behaving like an idealized polymer according to the worm-like-chain model. NC binds extended DNA through simple bimolecular binding, slightly shortening the polymer, and the NC-DNA complex relaxes as an idealized polymer with modified persistence length due to protein binding. Below a certain tensile force, NC immediately compacts the DNA, maintaining tension as extension reduces. The packaging rate was found to depend on the relaxation rate of the optical traps and was not limited to 1000 nm/s (Gien et al. 2022). In a different experiment, it was shown that once the NC-DNA condensate was formed, it was resistant to forces much above this force plateau.

Although DNA compaction by NC qualitatively resembled that of other multivalent cations, binding of NC to DNA was found to be ∼4 orders of magnitude larger than that of typical DNA-condensing cations cobalt hexamine (CoHex^3+^) and spermine (Spe^3+^), which is hypothesized to be partly due to aromatic stacking. In a later study with similar experiments, it was shown that there are specific cationic residues on NC that are important for DNA binding and condensation (Gien et al. 2024). The volume of dsDNA with associated NC molecules has been estimated to be similar to that of uncondensed, hydrated dsDNA (Burdick et al. 2024). Thus, in the DNA-NC condensate the volume of the NC molecules inside the capsid does not add to pressure built-up, which is suggested to lead to a relatively low pressure inside the capsid, protecting it against premature uncoating (Gien et al. 2022, Gien et al. 2024).

Optical tweezers were also exploited to study steps of the genome packaging process of SARS-CoV-2 (Morse et al. 2023). SARS-CoV-2 is an enveloped virus, where the genome inside is condensed by the nucleocapsid (N) protein (Eltayeb et al. 2024, Cubuk et al. 2021, Laughlin et al. 2024). The N protein consists of two important domains connected by a linker, the N terminal domain (NTD), thought to only participate in RNA binding, and the C-terminal domain (CTD), which also facilitates oligomerisation to form a nucleocapsid (Morse et al. 2023, Laughlin et al. 2024). By using optical tweezers, the authors directly measure the binding and genome condensing capacity of the SARS-CoV-2 nucleocapsid protein at the single-molecule level in real-time (Fig. 11a) (Morse et al. 2023). In this study, ssDNA was tethered between two beads with a small tension (~ 10 pN) to prevent complete collapse. Using full-length N (NFL) protein, two distinct nucleic acid compaction steps are observed with a pause in between. When incubating only with the NTD, it was found that NTD-DNA binding was non-cooperative bimolecular binding, and the DNA compaction was similar to that of the first compaction step observed with NFL protein. On the other hand, it was observed that the CTD does compact ssDNA fully, although slower than NFL. The resulting model that is proposed is based on these findings and suggests it follows a multistep process. The N protein exists in solution mainly as dimers in a high local concentration, as a result of viral gene expression. Binding with ssRNA occurs mainly through the NTD, followed by CTD mediated oligomerisation to encapsulate the genome. As this study was performed using an ssDNA substrate, while SARS-CoV-2 is an ssRNA virus, certain structures observed in WT virions may require interactions that were not observed in this study due to potentially specific or sequence-dependent interactions between vRNA and N protein.

The above studies demonstrate that protein-genome interactions responsible for compacting nucleic acids during viral packaging could be governed by common physicochemical forces. Electrostatic interactions between the negatively charged genome and positively charged capsid proteins primarily drive the assembly process (Buzón et al. 2020). These interactions are typically nonspecific, as is demonstrated by the ability of many systems capable of forming VLPs on different non-native polymers. For example, it was observed that SV40 capsid protein formed VLPs with several RNAs and even the synthetic polystyrene-sulfonate (PSS) (Li et al. 2017), despite natively being a DNA virus. The plant RNA virus cowpea chlorotic mottle virus (CCMV) was observed to package PSS preferentially over RNA, potentially due to PSS’s hydrophobic backbone (Tresset et al. 2014). It has been demonstrated that the template polymer affects the outcome of assembly (Li et al. 2017), but on mechanistic differences, not much is known. The observed nonspecific mechanics of viral packaging raise many questions regarding the assembly processes in vivo. The crowded nature of the host cellular environment requires a good degree of specificity. Besides the viral genome, many host nucleic acid molecules (such as mRNA) are present in the cytosol. The viral genome must be distinguished by the capsid protein so that only this genome is packaged into virions. Despite the observed non-specific interactions that drive self-assembly, viruses display a high degree of genome specificity, packaging only their native genome into infectious virions (Buzón et al. 2020, Bruijn et al. 2022).

The nature of this specificity is still debated and partially unclear. For some viruses it has been shown that they achieve specificity through so-called packaging signals, where the capsid proteins recognize short sequences on the genome that typically form some secondary structures displaying a high affinity for the capsid protein (Bruinsma et al. 2021, Buzón et al. 2020, Bruijn et al. 2022, Calcines-Cruz et al. 2021). Although it is generally appreciated that packaging signals impact assembly, their effect on the kinetics of assembly is not well-known. An artificial packaging signal based on CRISPR-Cas proteins was used together with the synthetic polypeptides to emulate a packaging signal in an artificial system (Marchetti et al. 2019, Calcines-Cruz et al. 2021). Based on the observations in this study, a kinetic model for genome packaging with artificial packaging signals was developed (Bruijn et al. 2022). It was found that the Cas proteins could act as efficient nucleation points, dramatically increasing the packaging rate. Template polymer effects, including the effect of packaging signals, have not yet been studied in detail using optical tweezers. Combining an optical tweezers experiment with high-resolution fluorescence techniques, potentially even super-resolution microscopy, could reveal interesting details about preferential localization of the capsid proteins on the substrate. Studying packaging signals with optical tweezers does provide challenges. For example, the packaging signal is often located at the end of the nucleic acid substrate, where the substrate should be attached to the beads via a biotin-streptavidin bond. In addition, WT viral RNA often displays a complex secondary structure, which is impractical because an optical tweezers experiment often requires an extended tether. A solution to this problem could be provided by the use of DNA handles, where an RNA molecule is covalently bound to DNA molecules attached to the beads. Such an approach allows for a stable tether of secondary RNA structures in a dual optical trap. This strategy was also used to investigate the dynamics of the TAR-RNA hairpin of HIV-1 (McCauley et al. 2015; Post et al. 2016) and the SARS-CoV-2 SL4 hairpin (Sundar Rajan et al. 2024). The artificial system could provide a good starting point for the study of packaging signals (Calcines-Cruz et al. 2021), as a similar system was already studied with optical tweezers (Marchetti et al. 2019), and it is compatible with linear dsDNA substrates.

## Conclusions

Unlike traditional bulk methods, optical tweezers - a laser-based tool capable of capturing and manipulating individual particles with pN forces - represent a relatively new approach in virology. The application of optical tweezers to study viruses is continually expanding. Optical tweezers can measure correlated parameters such as force-extension or torque-rotation over time, providing dynamic information that validates and refines previously hypothesized mechanistic models. This technology offers unparalleled insights into viral mechanisms.

Optical tweezers are a powerful single particle technique to study viral dynamics in real time. This technique has high spatial, temporal, and force resolution, probing biomolecular processes within their relevant range. Optical tweezers are extremely flexible in their experimental operation and allow for many different types of assays to be performed. Additionally, optical tweezers experiments are expanded with fluorescence microscopy to incorporate visual information with quantitative force data. The value of optical tweezers to single particle physical virology is demonstrated with their contributions to bacteriophage DNA packaging and self-assembly of virus-like particles on nucleic acid substrates. Developments towards increased reproducibility and resolution, in combination with advanced visualization techniques, will increase the potential of optical tweezers in the future. The impact of optical tweezers can even be increased by combining it with other techniques.

Optical tweezers experiments can for instance be combined with visual techniques such as fluorescence microscopy, electron microscopy, or (high speed) AFM. Performing simultaneously optical tweezers experiments and fluorescence microscopy imaging allows for the real time visualisation of processes while measuring forces on the same single molecular complex. Combining optical tweezers experiments with electron microscopy or AFM in one and the same experiment is more challenging. Typically these experiments are performed side by side on separately prepared samples and the data is then afterwards combined in order to provide an integrated picture of the observed processes. By combining optical tweezers experiments with HS-AFM experiments it has for instance been shown how deeper insights on viral assembly can be obtained (Buzón et al. 2021). Another example is the combination of high-resolution cryo-EM data with mechanistic insights obtained through optical tweezers, which together form a powerful system for uncovering the molecular mechanisms of various force-generating enzymes (Tong and Bustamante 2021). Information from optical tweezers also can be combined with traditional biochemical measurements and with molecular dynamics simulations to connect residue level chemistry to mechanochemical output, as demonstrated by studies that linked catalytic residues and arginine toggle behaviour to ATP hydrolysis and mechanochemical coupling (Ortiz et al. 2019). It would be interesting to study the packaging processes of other viruses, outside of dsDNA bacteriophages, using a similar combination of visualization and dynamic techniques. Furthermore, with asymmetric reconstructions, the conformations of nucleic acids inside viral particles can be elucidated to a much higher degree of precision (Beren et al. 2020). Combining these structures with optical tweezers experiments probing mobility of DNA inside capsids and comparing these results with existing theories on soft matter systems will likely increase our understanding of the physics associated with tight DNA confinement (Fizari et al. 2023). Studying the effects of template polymers and packaging signals on the mechanics of virion assembly may assist in the development of biologically relevant kinetic models for viral assembly. In addition, studying virus-like particles on synthetic poly-anions such as PSS provides interesting perspectives towards the development of virus-inspired systems as nanocontainers and delivery devices with polymer cargo.

There is a growing interest in the real-time characterization of viral mechanochemical functions, as well as in leveraging this knowledge for nanotechnological applications. In this review, we highlighted some of the key contributions of optical tweezers to the field of virology and present a comprehensive summary of studies on viral molecular dynamics, after integrating this dynamic information with structural and biochemical analyses, we can offer rigorous methods for modelling viral function and stability, enabling precise thermodynamic characterization of viral machinery. These models form the foundation for potential advancements in nanotechnology applications.
